# Draft Genome Sequence of Hyperthermophilic, Halotolerant Parageobacillus toebii PW12, Isolated from the Tattapani Hot Spring, Northwest Himalayas

**DOI:** 10.1128/MRA.01163-18

**Published:** 2019-01-24

**Authors:** Parul Sharma, Sonika Gupta, Anuradha Sourirajan, David J. Baumler, Kamal Dev

**Affiliations:** a Faculty of Applied Sciences and Biotechnology, Shoolini University, Solan, Himachal Pradesh, India; b Department of Food Science and Nutrition, University of Minnesota—Twin Cities, St. Paul, Minnesota, USA; c Microbial and Plant Genomic Institute, University of Minnesota—Twin Cities, St. Paul, Minnesota, USA; d Biotechnology Institute, University of Minnesota—Twin Cities, St. Paul, Minnesota, USA; Georgia Institute of Technology

## Abstract

Here, we report the genome sequence of hyperthermophilic and halophilic *Parageobacillus toebii* PW12, isolated from the Tattapani hot spring in the northwest Himalayas. The genome size of *Parageobacillus toebii* PW12 is 3,210,377 bp. The G+C content is 42.05%, and 3,382 coding sequences (CDS), 80 tRNAs, 5 noncoding RNAs (ncRNAs), and 4 CRISPR arrays were predicted.

## Abstract (precis)

Here, we report the genome sequence of hyperthermophilic and halophilic *Parageobacillus toebii* PW12, isolated from the Tattapani hot spring in the northwest Himalayas. The genome size of *Parageobacillus toebii* PW12 is 3,210,377 bp.

## ANNOUNCEMENT

Thermophilic microorganisms belonging to the Archaea and Bacteria inhabit hot springs, thermal pools, and fumaroles that are found throughout the world (1). Since the discovery of thermophilic microorganisms and novel enzymes, such as *Taq* polymerase from Thermus aquaticus, a large number of thermophiles have found industrial applications, including their importance as sources of thermostable enzymes (proteases, amylase, lipase, xylanase, cellulase, and DNA restriction enzymes) and other products of industrial utility (2–4). Geobacillus spp. were first classified by Nazina et al. (5) and constitute the most abundant thermophiles isolated from a wide range of environments (6), and they have huge biotechnological potential (7). Recently, polygenomic studies showed that the genus Geobacillus consists of two distinct genera, *viz.*, major Geobacillus and minor Parageobacillus (8). The objective of this study was to explore thermophilic bacteria from the Tattapani hot spring in the Himalayas that can be used for decomposition of cellulosic biomass in different polluted environments.

We isolated Parageobacillus toebii strain PW12 from a Tattapani hot spring water sample by spreading the water sample on nutrient agar medium and incubating it at 70°C (9). Isolated colonies were purified by the streaking method. The bacterial strain was identified by amplification and sequencing of 16S rRNA genes. The nucleotide sequence was subjected to a BLASTN (http://blast.ncbi.nlm.nih.gov) search of the NCBI database for taxonomic identity and submitted to NCBI under GenBank accession number KJ509869. The bacterial strain showed optimum cellulase enzyme activity at 80 to 90°C and pH 6 to 8.0, and cellulase activity was tolerant to metal ions (Mn^2+^, Co^2+^, Fe^2+^, Cd^2+^, and Hg^2+^), detergents (SDS, Triton X-100), solvents (toluene, cyclohexane, H_2_O_2_, *n*-butanol, ethanol), and EDTA (9). For genome sequencing, genomic DNA was extracted by a standard method for bacterial DNA isolation (10) and separated on 1% agarose gel by using TAE buffer (40 mM Tris base, 1 mM EDTA, and 20 mM glacial acetic acid) (10) and visualized by using a gel documentation unit (Alpha Innotech). A Qubit 2.0 fluorometer was used for determining DNA concentration. The paired-end (PE) sequencing library was prepared using an Illumina TruSeq Nano DNA high-throughput (HT) library preparation kit, and the PCR-amplified library was analyzed with a Bioanalyzer 2100 (Agilent Technologies) instrument using a high-sensitivity (HS) DNA chip, per the manufacturer's instructions, and loaded onto an Illumina HiSeq 2500 platform for cluster generation and sequencing. A total number of 1,707,006 paired-end reads with 512,101,800 bp was obtained using 2 × 150-bp chemistry on the Illumina platform. The *de novo* genome assembly of high-quality PE reads and scaffolding was accomplished using SOAPdenovo v.2 (11), with a genome coverage of 150.0×. In the Illumina sequencing, each base in a read with a Phred score of 30 was assigned a quality score by a Phred-like algorithm (12). The assembled genome sequence of Parageobacillus toebii PW12 consists of 3,238,931 bp, arranged into 232 scaffolds. The G+C content was 42.05%. Totals of 3,382 coding sequences (CDS), 80 RNAs, 5 noncoding RNAs (ncRNAs), and 4 CRISPR arrays were predicted using the National Center for Biological Information (NCBI) Prokaryotic Genome Annotation Pipeline and the best-placed reference protein set of GeneMarkS+ (Annotation Software v.4.6), as described (13, 14). Three trinucleotide [(CGC)_5_, (ATA)_5_, and (CTT)_5_] and two dinucleotide [(TA)_6_ and (CT)_6_] simple sequence repeats (SSRs) were identified using the MicroSatellite identification tool (MISA), as described (15). Genome statistics are summarized in Table 1.

**TABLE 1:** Global statistics of the genome sequence of Parageobacillus toebii PW12

Parameter	Statistic
Total sequence length (bp)	3,238,931
No. of genes (total)	3,462
No. of CDS (total)	3,382
No. of genes (coding)	3,006
No. of CDS (coding)	3,006
No. of RNA genes	80
Total no. (type) of rRNAs	2 (5S), 5 (16S), 5 (23S)
No. (type) of complete rRNAs	2 (5S)
No. (type) of partial rRNAs	5 (16S), 5 (23S)
No. of tRNAs	63
No. of ncRNAs	5
No. of CRISPR arrays	4
No. of scaffolds	232
Scaffold *N*_50_ (bp)	26,951
Scaffold *L*_50_	35
No. of contigs	570
Contig *N*_50_ (bp)	16,823
Contig *L*_50_	53

### Data availability.

This whole-genome shotgun project has been deposited at DDBJ/ENA/GenBank under the accession number QREZ00000000. The version described in this paper is version QREZ01000000. Raw sequence reads are available at NCBI under BioProject accession number PRJNA484024, BioSample accession number SAMN09756839 (Tattapani hot spring [Himachal Pradesh] isolate), and SRA accession numbers SRX5163167 (*Parageobacillus toebii* PW12 experiment) and SRR8352206 (Parageobacillus toebii_PW12_R1.fq.gz run).
